# Serum-Based Quantification of *MYCN* Gene Amplification in Young Patients with Neuroblastoma: Potential Utility as a Surrogate Biomarker for Neuroblastoma

**DOI:** 10.1371/journal.pone.0161039

**Published:** 2016-08-11

**Authors:** Shigeki Yagyu, Tomoko Iehara, Shiro Tanaka, Takahiro Gotoh, Akiko Misawa-Furihata, Tohru Sugimoto, Wendy B. London, Michael D. Hogarty, Satoshi Teramukai, Akira Nakagawara, Eiso Hiyama, John M. Maris, Hajime Hosoi

**Affiliations:** 1 Department of Pediatrics, Graduate School of Medical Science, Kyoto Prefectural University of Medicine, Kyoto, Japan; 2 Department of Pharmacoepidemiology, Graduate School of Medicine and Public Health, Kyoto University, Kyoto, Japan; 3 Division of Hematology/Oncology, Children's Hospital Boston/Dana-Farber Cancer Institute, Harvard Medical School, Boston, Massachusetts, United States of America; 4 Division of Oncology and Center for Childhood Cancer Research, Children’s Hospital of Philadelphia and University of Pennsylvania, Philadelphia, Pennsylvania, United States of America; 5 Department of Biostatistics, Graduate School of Medical Science, Kyoto Prefectural University of Medicine, Kyoto, Japan; 6 Division of Biochemistry and Innovative Cancer Therapeutics, Chiba Cancer Center, Chiba, Japan; 7 Saga Medical Cancer KOSEIKAN, Saga, Japan; 8 Department of Pediatric Surgery, Hiroshima University Hospital, Natural Science Center for Basic Research and Development, Hiroshima University, Hiroshima, Japan; Gustave Roussy, FRANCE

## Abstract

We previously developed a method for determining *MYCN* gene amplification status using cell-free DNA fragments released from cancer cells into the blood of patients with neuroblastoma (NB). Here, we analyzed the relationship between *MYCN* amplification (MNA) status and neuroblastoma prognosis. We screened serum samples from 151 patients with NB for MNA, using real-time quantitative PCR, and compared the results with *MYCN* status determined using paired tumor samples. We additionally investigated whether MNA status correlates with patient survival. When a cut-off value of 5 was used, serum-based MNA analysis was found to show good sensitivity (86%) and very high specificity (95%). The sensitivities for stage 1 and 2 might be acceptable, even though it is not as good as for stage 3 and 4 (67% for stage 1 and 2, 92% for stage 3, and 87% for stage 4). MNA status correlated with overall survival in our cohort of 82 patients, with survival data available (p < 0.01). The hazard ratio of MNA status was 4.98 in patients diagnosed at less than 18 months of age (95% confidence interval, 1.00–24.78), and 1.41 (95% confidence interval, 0.63–3.14) for those diagnosed at 18 months of age or older. Serum-based MNA analysis is rapid and non-invasive compared with tumor-based MNA analysis, and has potential to predict tumor MNA status. There is still a room to improve the sensitivity of the test for tumors of stages 1 and 2, nonetheless this assay might help to determine therapeutic strategies prior to tumor biopsy, especially for patients with a life-threatening condition, as well as for patients of less than 18 months of age whose risk-grouping and treatment allocation depends on their MNA status.

## Introduction

Patients with neuroblastoma (NB) fall into 2 clinically distinct subgroups: a low-risk subgroup and a high-risk subgroup based on their age of onset [1], the extent of the disease [2], pathological findings [3], and genomic changes in NB tumors. The prognosis of NB depends on the age of onset; in addition, most NB tumors produce catecholamine metabolites. Therefore, a screening test for NB has been designed with the aim of enabling early intervention and consequent improvement in survival in patients with NB. This nationwide screening system, which was launched in Japan in 1973 and later introduced in other countries [4, 5], has facilitated the detection of NB in numerous patients of less than one year of age prior to the overt clinical manifestation of symptoms. Consequently, the diagnosis rates of NB sharply rose after the screening commenced; however, most patients diagnosed with NB via screening exhibited good prognosis [6, 7]. Further observation revealed that most of the subclinical tumors were occult tumors capable of spontaneously regressing or differentiating without becoming clinically overt [8–10]. Moreover, 2 prospective studies concluded that detection of subclinical NB by mass screening did not reduce overall mortality in patients with this disease [11, 12]. Therefore, mass screening for NB was criticized as being “over-diagnostic,” and the Japanese nationwide screening program for the early detection of NB was paused in 2004. Recent efforts have focused on the development of novel, clinically useful, and cost-effective NB screening systems to enable improvements in outcome for patients with NB.

In contrast to the above findings, those of a population-based cohort study assessing the effectiveness of the Japanese mass screening program revealed that the mortality rates of children with NB, who were diagnosed via screening at the age of 6 months, were lower than in the prescreening cohort [13]. These data imply that certain high-risk patients with NB may benefit from early diagnosis (via screening) and intervention. Therefore, the development of a method for identification of such patients within the screened cohort is desirable for improving the effectiveness of screening programs.

The most powerful genetic prognostic factor in NB is *MYCN* gene amplification (MNA) status. We, and others, have demonstrated that MNA is strongly correlated with poor prognosis even in infantile NB [14, 15], and that the outcomes of patients with NB that exhibit MNA are significantly worse than those that do not exhibit MNA NB patients, even in patients aged less than 18 months [14, 15]. Therefore, for patients diagnosed with NB at less than 18 months of age, rapid and accurate determination of MNA status is essential.

We previously developed a detection system for tumor MNA status using tumor-derived DNA fragments extracted from patients’ sera [16]. The present study aimed to investigate the utility of MNA status as a tool for the identification of high-risk patients with NB that may benefit from early therapeutic intervention. We attempted to determine whether the previously developed serum-based MNA analysis enables the identification of patients with poor prognosis among infantile cases in general. In addition, we investigated whether the detection of *MYCN* DNA in serum, in addition to the measurement of urine catecholamine metabolites, may be used as a secondary screening tool for infantile NB.

## Materials and Methods

### Subjects

We collected tumor and serum samples as well as clinical data for patients registered with tissue banks at the Children’s Oncology Group in the USA, and Hiroshima University and the Chiba Cancer Center Research Institute, both in Japan. Eligibility criteria were: 1) histologically confirmed NB diagnosis, 2) evaluation of MNA status by Southern blotting or interphase fluorescence in situ hybridization, 3) availability of tumor and serum samples, and 4) provision of written informed consent of patients or their parents at the time of sample collection. Data management was conducted by an independent data center. The serum and tumor samples were linked with clinical data at the data center and the laboratory-based investigators were blinded to these data. This study was conducted in accordance with the Declaration of Helsinki and the research protocol was approved by the Kyoto Prefectural University of Medicine ethical review committee.

**Fig 1:**
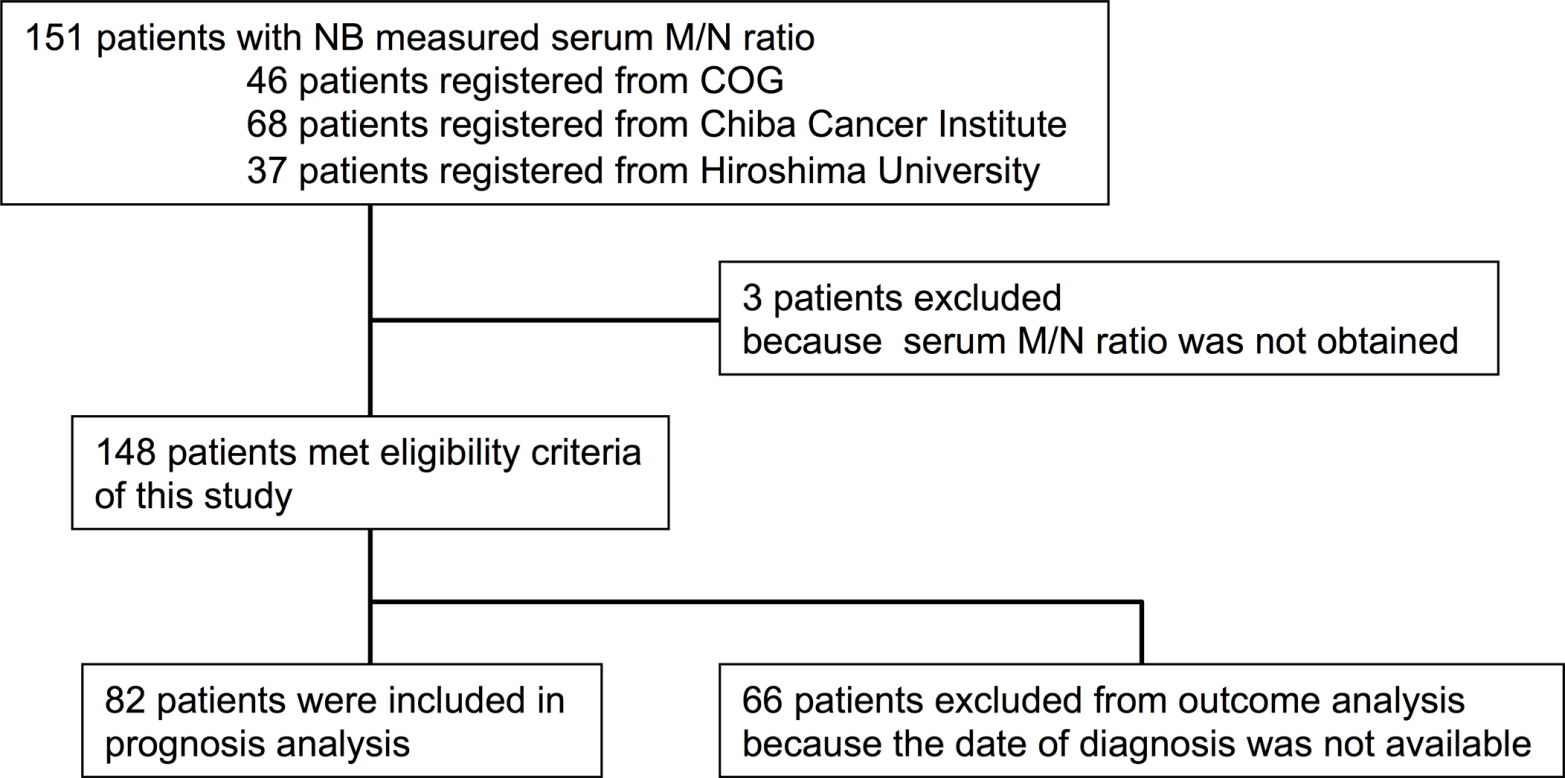
Patient disposition.

A total of 151 tumor and serum samples were collected; 37 from patients registered at Hiroshima University between 1983 and 2002, 68 from patients registered at Chiba Cancer Center Research Institute between 2001 and 2006, and 46 from patients registered at the Children’s Oncology Group between 2000 and 2006 (Fig 1). The date of diagnosis was not available for patients registered at the Chiba Cancer Center Research Institute. The ratios of *MYCN* and *NAGK* gene dosage (M/N ratios) [16] in 3 of the 151 patients could not be evaluated as no *MYCN* or *NAGK* amplicon was detected in samples from these patients. In addition, overall survival of 66 of the 148 patients, whose M/N ratio could be evaluated, could not be determined. Therefore, 82 patients were finally included in the study for determination of overall survival. The MNA status of the tumors was analyzed at each respective institute.

### DNA extraction from tumor and serum samples

Tumor DNA was extracted at each institute according to the standard protocol, stored at -20°C, and used for this study under the approval of the relevant Institutional Review Boards. The serum samples were obtained from patients prior to therapy or surgery, and were stored at -20°C. Serum DNA was extracted as described previously [16, 17].

### Real-time quantitative PCR

Circulating *MYCN* gene dosage was quantitated by real-time quantitative PCR, as previously described [16]. The *MYCN* copy number of a sample of DNA was determined as the ratio of *MYCN* dosage to *NAGK* dosage (M/N ratio). Copy numbers were expressed as the average of 2 measurements.

### Statistical considerations

Planned sample size and all statistical analyses were pre-specified in the research protocol. The planned sample size of 170 was set to obtain sensitivity and specificity narrower than ± 5% with 95% confidence intervals (CIs), based on our previous study [16].

The primary endpoint was overall survival time, defined as the time from diagnosis to death. Additionally, the diagnostic accuracy of the serum M/N ratio as a predictor of tumor MNA status was evaluated. The difference in serum M/N ratio between the MNA and non-MNA groups was assessed using the Wilcoxon rank sum test. Spearman’s rank correlation was calculated to assess the correlation between serum and tumor M/N ratio. In order to identify the optimal cut-off point for the M/N ratio, a receiver operating characteristic (ROC) curve was constructed and the area under the curve (AUC) was calculated. To determine overall survival time, survival curves were derived by the Kaplan-Meier method. Performance of the serum M/N ratio as a prognostic marker was evaluated by multivariate Cox proportional hazards models. Age and tumor stage were specified in the research protocol as adjustment and stratification factors. All the missing data were treated by complete-case analysis. Two-sided p values of less than 0.05 were considered to represent statistical significance.

## Results

### Patient characteristics

Table 1 describes the clinical characteristics of the 148 eligible patients. At the time of diagnosis, 31 patients were younger than 18 months, 49 were between 18 months and 16 years of age, and the date of diagnosis or birth date was not available for 68 patients of Chiba Cancer Center Research Institute, as mentioned above. Fifty-seven of the patients exhibited MNA, and 91 patients did not exhibit MNA.

**Table 1:** Patient characteristics.

Characteristics		No. of patients	Mean	SD
Age of diagnosis (day)			1064.9	1078.3
Less than 18 months		31		
Over 18 months		49		
Data not available		68		
Stage (INSS)	1	29		
	2	4		
	2a	8		
	2b	3		
	3	26		
	4	71		
	4s	7		
MYCN status	Amp.	57		
	non-Amp	91		

### Serum M/N ratio as a predictor of tumor MNA status

The serum M/N ratio of the MNA group (n = 57, median M/N ratio = 118.27) was significantly higher than that of the non-MNA group (n = 91, median M/N ratio 2.45; p < 0.01; S1A Fig). These results confirm those of our previous small-scale study [16]. The ROC curve (S1B Fig) indicates that the serum M/N ratio has excellent diagnostic power. When the cut-off value for the serum M/N ratio was 5, the AUC was 0.911 (95% CI; 0.849–0.974), and the sensitivity and specificity were 86% (95% CI; 74–94%) and 95% (95% CI; 88–98%), respectively. Moreover, the serum M/N ratios were well correlated with the tumor M/N ratios (Spearman correlation coefficient = 0.671, 95% CI; 0.570–0.751).

Next, we examined the differences in sensitivity and specificity of this assay between International Neuroblastoma Staging System (INSS) stages. A subgroup analysis according to INSS stage revealed no statistically significant differences in sensitivities and specificities; however, the low statistical power, owing to the small number of patients in each group, did not enable the detection of differences (p = 0.48 for sensitivity, p = 0.68 for specificity, chi-squared test, Table 2). These data demonstrate that the serum M/N ratio is a specific tool for the prediction of tumor MNA status in patients with NB, regardless of tumor stage, though the sensitivity in stage 1/2 NB showed lower than in stage 3 or 4 NB.

**Table 2:** Diagnostic accuracy of serum M/N ratio according to stage.

			Non-amplified tumor	Amplified tumor	Sensitivity (95% CI)	Specificity (95%CI)
Stage (INSS)		Serum M/N ratio				
	Stage 1 and 2	less than 5.0	36	2	67%	95%
		over 5.0	2	4	(22–96%)	(82–99%)
	Stage 3	less than 5.0	12	1	92%	86%
		over 5.0	2	11	(62–100%)	(57–98%)
	Stage 4	less than 5.0	32	5	87%	97%
		over 5.0	1	33	(72–96%)	(84–100%)
	Stage 4s	less than 5.0	6	0	100%	100%
		over 5.0	0	1	(3–100%)	(54–100%)
Total			91	57	86%	95%
					(74–94%)	(88–98%)

CI: Confidence interval

### Serum M/N ratio as a prognostic marker for NB in patients less than 18 months of age

**Fig 2:**
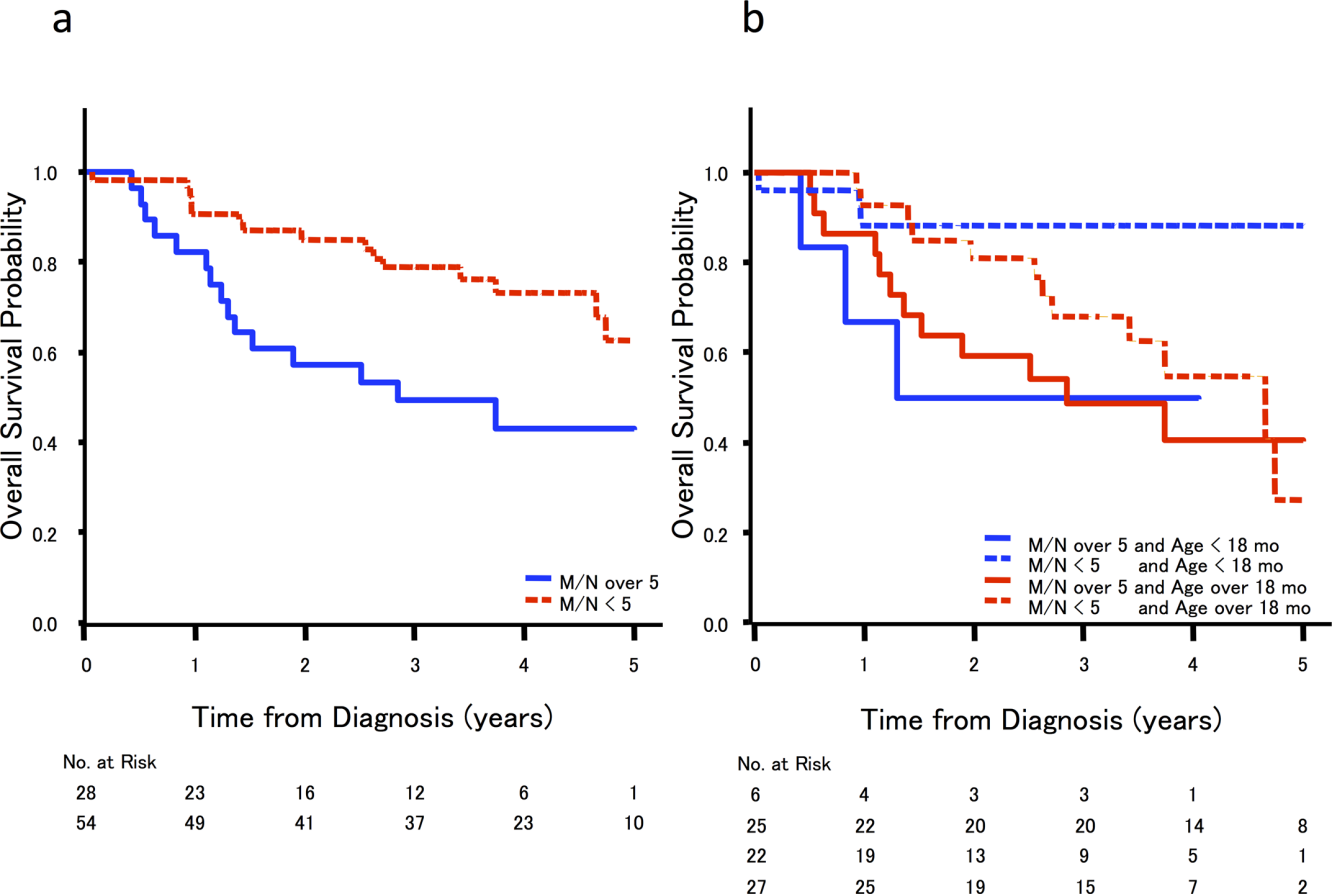
**Kaplan-Meier survival curves for 82 patients and correlation between (a) serum M/N ratio and age of diagnosis and (b) serum M/N ratio and overall survival rate**.

The correlation between serum M/N ratio and clinical outcome was analyzed in 82 cases for which clinical data were available. As anticipated by the high sensitivity of this serum based MNA analysis, univariate analyses revealed that patients with MNA, as determined based on their serum M/N ratio, exhibited significantly worse overall survival than patients without MNA (hazard ratio: 2.50; 95% CI; 1.22–5.13, p < 0.01, Fig 2A). A subgroup analysis revealed that serum M/N ratio also had significant prognostic power when limited to cases diagnosed at less than 18 months of age. However, in the subset of patients older than 18 months, we did not observe a significant difference in survival between patients with and without MNA (Fig 2B, Table 3); these findings were consistent with those of previous studies of the prognostic power of tumor MNA status [18]. These data suggest that serum M/N ratio may be utilized as a surrogate prognostic marker for NB in patients less than 18 months of age.

**Table 3:** Subgroup analysis for prognostic impact of serum M/N ratio according to age of diagnosis.

		No. of patients	Hazard ratio	95% CI		p-value	Interaction p-value
Age less than 18 months							
	Serum M/N ratio less than 5.0	25	1	-	-	-	-
	Serum M/N ratio over 5.0	6	4.98	1.00	24.78	0.05	-
Age over 18months							
	Serum M/N ratio less than 5.0	27	1	-	-	-	-
	Serum M/N ratio over 5.0	22	1.41	0.63	3.14	0.41	
							0.08

CI = confidence interval

Two patients were excluded as their ages were not known.

## Discussion

Our results show that serum-based analysis of MNA has sufficiently high sensitivity and remarkably high specificity for detecting the status of amplification of the *MYCN* gene in serum samples. The serum M/N ratio of patients with NB has considerable prognostic value, especially in patients aged less than 18 months, confirming that MNA status is useful as a prognostic biomarker even in infantile NB [14, 15]. Previous nationwide mass screening programs for NB have revealed that most cases of localized infantile NB showed good prognosis [5–7, 11, 12] and that de-escalated therapies were justified in such patients. Even after suspension of screening in Japan in 2004, physicians currently recommend conservative therapies, including surgery alone or even expectant observation, following tumor biopsy in children with NB diagnosed at less than one year of age [19–21]. Although this expectant observation strategy was initially applied to patients with perinatally detected adrenal masses, this strategy is now being extended to other young patients with localized NB [21]. However, this approach may lead to misclassification of patients, especially in infantile cases with MNA-associated NB, and precise evaluation of prognostic factors using tumor markers obtained by less invasive techniques are desirable for such patients. The previously developed serum assay should be useful for evaluation of prognosis in this group. In addition to the disease stage, we propose that serum MNA status should be evaluated in newly diagnosed infantile patients with NB exhibiting high urinary levels of catecholamine. In patients with NB, who exhibit localized tumors and serum M/N ratios of less than 5, a good prognosis is likely and expectant observation would be justified. However, we would recommend that patients whose serum M/N ratios are higher than 5, even infants with localized tumors, undergo tumor biopsy to determine the precise risk classification.

In patients older than 18 months, we did not observe significant difference in survival between patients with and without MNA, although there was no deviation in MNA status in patients older than 18 months. In these older patients, the tumor may have acquired genetic aberrations besides MNA that increased the likelihood of a poor outcome [22–28]. We previously developed a serum-based assay system for the detection of promoter methylation status or chromosomal loss of heterozygosity, both of which are associated with poor prognosis in NB [17, 29].

In the present cohort, 5 of the 57 positive cases were false positives and 8 of the 91 negative cases were false negatives. In 3 of the 5 false-positive cases, in which the serum M/N ratio appeared to be high, the MNA status of the tumor was negative. Further, the serum M/N ratios were well above 5 in 3 of the false positive cases (data not shown). Although the reason for this discrepancy is unknown, it may be attributable to the normal biological heterogeneity of NB tumors and/or an insufficient number of tumor samples. Therefore, we propose that, when the serum M/N ratio of a patient with non-MNA-associated NB is determined to be high, the serum M/N ratio should be carefully re-evaluated, or the biopsy sample should be re-examined for sufficient amount of tumor tissue or homogeneity.

In the 8 false-negative cases, in which the serum M/N ratio was below 5, the MNA status of the tumor was positive. This represents a serious limitation in the potential utility of serum M/N ratio as a prognostic marker, as false-negative results may lead to therapeutic abstention in patients with MNA-positive tumors. A possible reason for this discrepancy is that the sensitivity of serum-based determination of MNA status may be lower in patients with locoregional NB [30]. Indeed, our data suggest a lower sensitivity for stages 1 and 2 (Table 2), although the number of patients in each group did not allow sufficient statistical power to detect significant differences. The lower sensitivity in patients with locoregional NB may be attributable to (1) the smaller quantity of DNA fragments released from a small tumor burden, (2) an apparent reduction in M/N ratio due to tumor heterogeneity, or (3) low-quality serum samples in which the DNA stability is affected. We speculate that one of the most likely reasons for low serum M/N ratios in the present study was poor quality of the serum samples, which represents the major limitation of this retrospective study. The collected sera were not always processed immediately, and therefore may have become contaminated with DNA fragments from lysed leukocytes. Contamination of serum with DNA from normal tissue dilutes tumor-derived DNA fragments and reduces the serum M/N ratio [16]. Indeed, DNA fragments from leukocytes may have been released during storage, contaminating the serum sample and affecting the accuracy of measurement of serum M/N ratios (S3 Fig). In order to determine the serum *MYCN* status accurately, contamination due to the presence of normal cells/tissue should be avoided by carefully removing these cells from the serum by high-speed centrifugation or filtration as soon as possible after collection of the blood sample [16]. Moreover, sera must be handled promptly and appropriately and banked using a standardized method; of the 49 samples that were promptly and appropriately handled, both sensitivity and specificity of detection of tumor MNA status was 100% (S2 Fig). We believe that technical progress, for example a highly effective method to purify the tumor-released DNA fragments from serum and to keep the stability of the DNA fragments in serum, could be made regarding this problem.

A partial discrepancy was observed between the findings of our previous study [30] and the present data, particularly with regard to the sensitivity of serum MNA status in patients with low-stage NB. Combaret and colleagues, who first identified the *MYCN* gene in the sera of patients with NB, retrospectively analyzed the serum M/N ratios using stored serum samples from such patients. Their findings showed that the sensitivity of serum MNA status depends on the NB stage and is lower for patients with locoregional NB [30]. The authors analyzed 10 serum samples obtained at diagnosis from patients with MNA-associated NB at INSS stages 1 or 2, and 16 serum samples from patients with MNA-associated NB at INSS stage 3. They revealed that only one of 10 patients with stage 1 or 2 disease, and 12 of the 16 patients with stage 3 disease showed high levels of circulating *MYCN* DNA (10% and 75% sensitivity, respectively). In contrast, our data indicated a sensitivity of 67% for detection of patients with stage 1/2 NB. As both studies used the same detection method [16] and incidentally used the same cut-off value of 5, this discrepancy may be attributed to differences in the amount and purity of *MYCN* sequences retrieved from the serum of patients with stage 1/2 disease. The concentration of DNA fragments in the sera of patients with metastatic tumors was significantly higher than in those of patients with localized tumors [31], although tumor-derived cell-free DNA was also found in the serum of patients with localized, early-stage tumors [16, 17, 32]. Therefore, the degradation of serum DNA or contamination with DNA fragments from normal tissue would exert a greater impact on accuracy of diagnosis of locoregional NB than on metastatic NB. The translation of our findings into the clinic would require the development of specialized devices or kits in order to reduce levels of degradation or contamination resulting from the presence of tumor-derived DNA fragments in serum samples, and for improved sensitivity of detection of MNA status. The additional use of other serum-based biomarkers, such as loss of 11q or aberrant DNA hypermethylation, in combination with the present method, may be useful for accurate identification of high-risk patients with NB.

## Conclusion

The application of the present findings to population-screening programs requires improvement in the sensitivity of the detection method especially for the patients with low-stage NB; however, the present assay should be useful for the design of therapeutic strategies for patients with NB prior to a tumor biopsy, especially for patients with a life-threatening condition and those less than 18 months of age, whose risk grouping and treatment allocation depends on their MNA status. The advantages of this method for preoperative evaluation of MNA status are that it (1) benefits patients with NB, including children, by enabling rapid determination of MNA status prior to biopsy or surgical resection, (2) involves no procedures that are laborious for pediatric surgeons and oncologists or hazardous for patients, and (3) requires only 200 μL of serum and approximately 4 h to analyze, making it a relatively low-cost procedure suitable for all patients with high concentrations of urine catecholamine metabolites. Screening strategies based on a combination of serum-based evaluation of MNA status and other biological assays, which would compensate for “false negative” cases of diagnosis via serum-based analysis of MNA status alone, represent a potentially useful next-generation screening tool for identification of high-risk patients with NB, even in infantile cases.

## Supporting Information

S1 Fig Sensitivity and specificity of serum M/N ratio. (a) Box plot of serum M/N ratio in patients with *MYCN* amplification (MNA)- and non-MNA-associated neuroblastoma. The serum M/N ratio was significantly higher in the MNA group (n = 57; median M/N ratio: 118.27; range: 1.09–3889.17) than in the non-MNA group (n = 91; median M/N ratio: 2.45; range: 0.63–129.23; p < 0.01, Mann-Whitney U test). (b) Receiver operating characteristic curve for sensitivity and specificity of serum M/N ratio: the area under the curve was 0.911 (95% confidence interval (CI); 0.849–0.974), and the sensitivity and specificity were 86% (95% CI; 74–94%) and 95% (95% CI; 88–98%), respectively, when the cut-off value for the serum M/N ratio was 5.
(PDF)

S2 Fig Serum M/N ratios measured immediately after serum collection. Sera of 59 patients with neuroblastoma were collected prior to tumor biopsy. Subsequently, contaminating white blood cells were immediately removed to prevent their interference with determination of DNA-based *MYCN* amplification (MNA) status. The serum *MYCN*/*NAGK* (M/N) ratio was assessed prospectively for all cases; when the ratio was 5 or greater, this was interpreted as MNA-positive. Following the determination of serum M/N ratio, each biopsied tumor sample was assessed by interphase fluorescence in situ hybridization (I-FISH). Among the 59 cases, 10 cases could not be evaluated due to lack of tumor samples. In the other 49 cases, in which tumor *MYCN* status could be evaluated, 13 cases exhibited MNA and 36 cases did not. Serum M/N status of all 13 MNA cases was greater than 5.0, while that of the other 36 non-MNA cases were less than 5.0.
(PDF)

S3 Fig Contamination of DNA fragments from lysed leukocytes. Leukocytes (5 × 10^3^) were added to 200 μl of IMR32 NB cell (*MYCN* gene amplified) culture supernatant and stored at 4°C. Then, leukocytes were removed from the samples by centrifugation (15000rpm, 10min at 4°C) immediately as well as after 1, 4, 7, and 14 days, and the M/N ratios were calculated. For leukocytes removed immediately from the supernatant, the M/N ratio was almost equivalent to that of non-contaminated samples, whereas the M/N ratio was greatly reduced in samples stored, without leukocyte removal, for over 4 days.
(PDF)
