# Supplementary figures and images for: Serum-Based Quantification of MYCN Gene Amplification in Young Patients with Neuroblastoma: Potential Utility as a Surrogate Biomarker for Neuroblastoma
Source: PLoS One. 2016 Aug 11;11(8):e0161039. doi: 10.1371/journal.pone.0161039 (PMC4981470; doi:10.1371/journal.pone.0161039)

S1 Fig

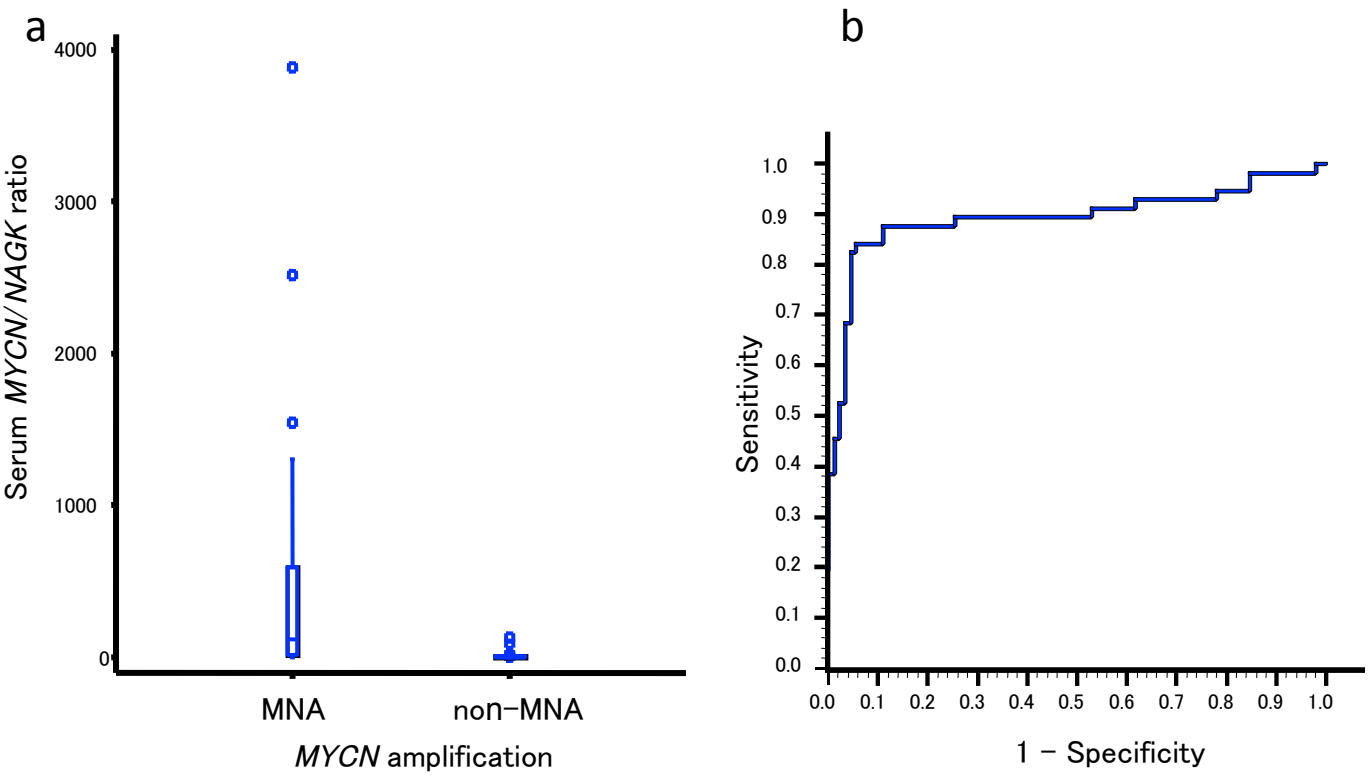

Supplement: S1 Fig — (a) Box plot of serum M/N ratio in patients with MYCN amplification (MNA)- and non-MNA-associated neuroblastoma. The serum M/N ratio was significantly higher in the MNA group (n = 57; median M/N ratio: 118.27; range: 1.09–3889.17) than in the non-MNA group (n = 91; median M/N ratio: 2.45; range: 0.63–129.23; p < 0.01, Mann-Whitney U test). (b) Receiver operating characteristic curve for sensitivity and specificity of serum M/N ratio: the area under the curve was 0.911 (95% confidence interval (CI); 0.849–0.974), and the sensitivity and specificity were 86% (95% CI; 74–94%) and 95% (95% CI; 88–98%), respectively, when the cut-off value for the serum M/N ratio was 5. (PDF) [file pone.0161039.s001.pdf]

S2 Fig

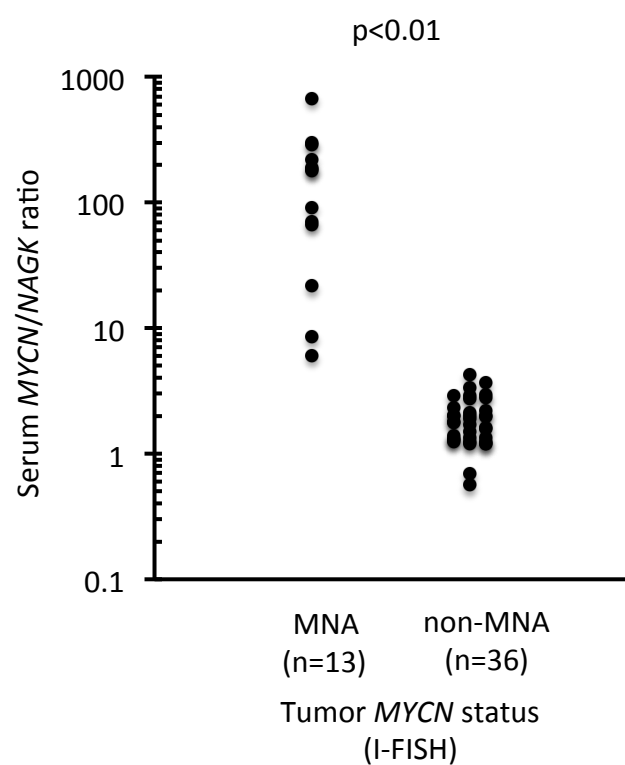

Supplement: S2 Fig — Sera of 59 patients with neuroblastoma were collected prior to tumor biopsy. Subsequently, contaminating white blood cells were immediately removed to prevent their interference with determination of DNA-based MYCN amplification (MNA) status. The serum MYCN/NAGK (M/N) ratio was assessed prospectively for all cases; when the ratio was 5 or greater, this was interpreted as MNA-positive. Following the determination of serum M/N ratio, each biopsied tumor sample was assessed by interphase fluorescence in situ hybridization (I-FISH). Among the 59 cases, 10 cases could not be evaluated due to lack of tumor samples. In the other 49 cases, in which tumor MYCN status could be evaluated, 13 cases exhibited MNA and 36 cases did not. Serum M/N status of all 13 MNA cases was greater than 5.0, while that of the other 36 non-MNA cases were less than 5.0. (PDF) [file pone.0161039.s002.pdf]

Supplemental Figure 3

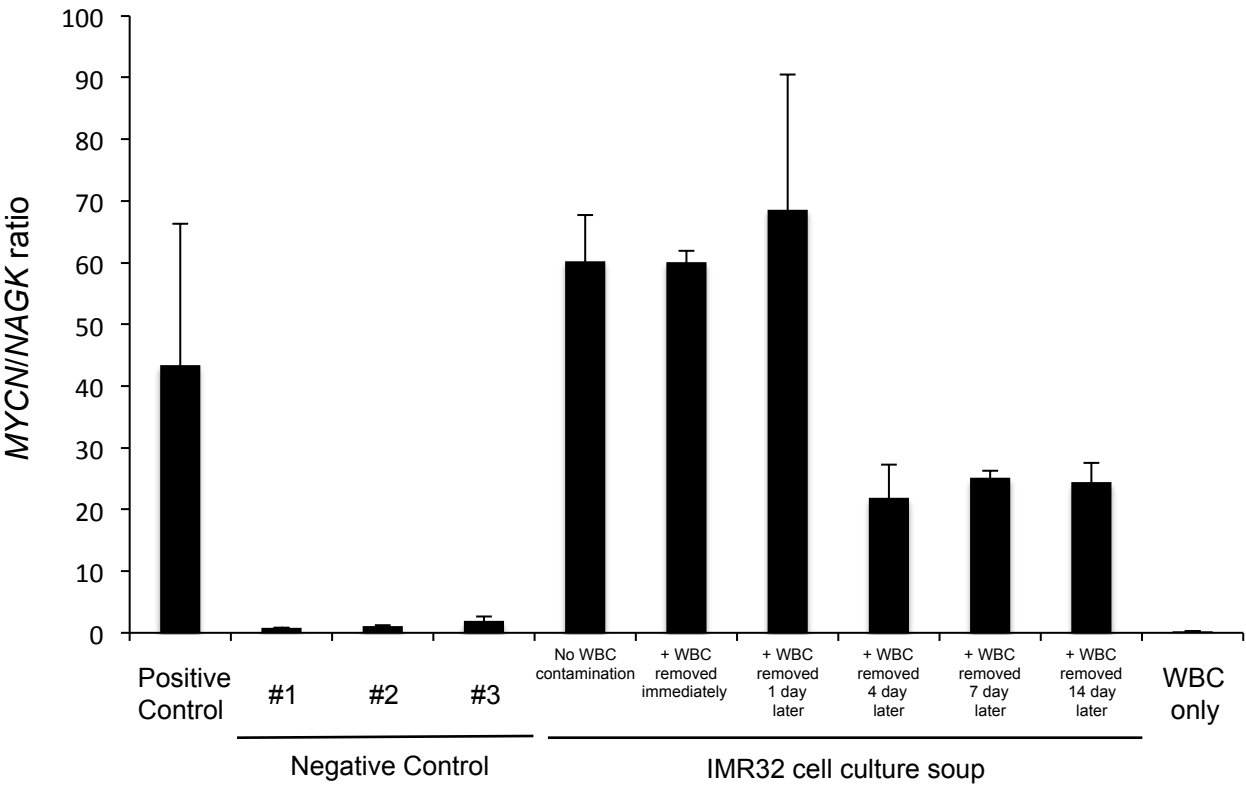

Supplement: S3 Fig — Leukocytes (5 × 10^3^) were added to 200 μl of IMR32 NB cell (MYCN gene amplified) culture supernatant and stored at 4°C. Then, leukocytes were removed from the samples by centrifugation (15000rpm, 10min at 4°C) immediately as well as after 1, 4, 7, and 14 days, and the M/N ratios were calculated. For leukocytes removed immediately from the supernatant, the M/N ratio was almost equivalent to that of non-contaminated samples, whereas the M/N ratio was greatly reduced in samples stored, without leukocyte removal, for over 4 days. (PDF) [file pone.0161039.s003.pdf]
